# Decoupling Intensity Radiated by the Emitter in Distance Estimation from Camera to IR Emitter

**DOI:** 10.3390/s130607184

**Published:** 2013-05-31

**Authors:** Angel E. Cano-García, José Luis Lázaro Galilea, Pedro Fernández, Arturo Luis Infante, Yamilet Pompa-Chacón, Carlos Andrés Luna Vázquez

**Affiliations:** 1 Communication Departament, Electrical Engineering College, University of Oriente, Av. Las Américas, Santiago de Cuba 90100, Cuba; E-Mails: angelcano78@gmail.com (A.E.C.-G.); ainfante@fie.uo.edu.cu (A.L.I.); ypompa@fie.uo.edu.cu (Y.P.-C.); 2 Electronics Department, Superior Polytechnic School, University of Alcalá, University Campus, Alcalá de Henares 28871, Madrid, Spain; E-Mails: pedro.fernandez@depeca.uah.es (P.F.); caluna@depeca.uah.es (C.A.L.V.)

**Keywords:** distance estimation sensor, radiometric, camera-to-emitter distance estimation, computer vision

## Abstract

Various models using radiometric approach have been proposed to solve the problem of estimating the distance between a camera and an infrared emitter diode (IRED). They depend directly on the radiant intensity of the emitter, set by the IRED bias current. As is known, this current presents a drift with temperature, which will be transferred to the distance estimation method. This paper proposes an alternative approach to remove temperature drift in the distance estimation method by eliminating the dependence on radiant intensity. The main aim was to use the relative accumulated energy together with other defined models, such as the zeroth-frequency component of the FFT of the IRED image and the standard deviation of pixel gray level intensities in the region of interest containing the IRED image. By using the abovementioned models, an expression free of IRED radiant intensity was obtained. Furthermore, the final model permitted simultaneous estimation of the distance between the IRED and the camera and the IRED orientation angle. The alternative presented in this paper gave a 3% maximum relative error over a range of distances up to 3 m.

## 1. Introduction

The camera model used in computer vision involves a correspondence between real world 3-D coordinates and image sensor 2-D coordinates by modeling the projection of a 3-D space onto the image plane [1]. This model is commonly known as the *projective model*.

If a 3-D positioning system is implemented using one camera modeled by the projective model, this would imply relating the image coordinate with the world coordinate, obtaining an ill-posed mathematical problem. In this analysis, one of the three dimensions is lost. From a mathematical point of view, this implies that although the positioning system is capable of estimating a 2-D position of the subject, the distance between the camera and the subject cannot be calculated. In fact, the position in this case would be defined by a line passing through the optical center and the 3-D world coordinates of the subject.

The main problem in this case can thus be defined: how can the distance between the camera and a subject be estimated efficiently? That is, how can depth be estimated?

Mathematically, the typical projective model can be used to estimate depth by including additional constraints in the mathematical equation system. These additional constraints could be incorporated, for example, by using another camera to form a stereo vision system [1–4], by camera or subject motion [5], by structured light patterns emitted to the environment [6], by focus-defocus information [7], and more recently, by a radiometric approach considering certain specific conditions [8–12].

The radiometric model and the alternative distance measurement method proposed in [8–12] could present a practical solution to depth estimation to ensure 3-D positioning, because they are independent of image coordinates and only use information extracted from pixel gray level intensities, which is not used for absolute measurement.

### 1.1. Previous Distance Measurement Alternatives

In the image formation process, the camera accumulates incident light irradiance during exposure time. The accumulated irradiance is converted into an electrical signal that is sampled, quantized and coded to form pixel gray level intensity. By analyzing this process inversely, from pixel gray level intensity to the irradiance on the sensor surface, an inverse camera response function can be defined, and this constitutes the starting point for applying a radiometric model [13,14].

The function that relates pixel gray level intensities to light irradiance on the sensor surface is known as the camera radiometric response function [13,14], and it is commonly used to produce high dynamic range images.

However, in [8,9,11], the inverse camera radiometric response function was used to extract a measure of the irradiance accumulated by the camera in the image formation process, in order to then use this as an empirical parameter to extract depth information under specific conditions.

For example, if the positioning system is designed to estimate the 3-D position of a robot carrying an infrared emitter diode (IRED), then the IRED can be considered the point-source and the irradiance on the sensor surface (camera) will decrease with squared distance [15]. Therefore, this is the main hypothesis that has been used to define a radiometric model oriented to the distance estimation tasks in [8,9,11], which is based on the following conditions:

- Images must be formed by the power emitted by the IRED. Background illumination must be eliminated using interference filters.
- The IRED must be biased with a constant current to ensure a constant radiant intensity.
- Focal length and aperture must be constant during the measurement process, as stated in projective models.
- The camera and the IRED must be aligned. The orientation angles have not been considered yet.
- The camera settings (such as gain, brightness, gamma) must be established in manual mode with a fixed value during the experiment. The goal is to disable any automatic process that could affect the output gray level.

Obviously, the power emitted by the IRED is affected by the azimuth and elevation angles. It can be modeled by the IRED emission pattern. As was stated in the conditions defined above, in a previous experiment, the camera was aligned with the IRED. However, in this paper the effect of the emission pattern of the IRED would be considered.

Another alternative to decrease the effect of the IRED emission pattern could be the use of an IRED source with an emission pattern conformed by optical devices. The idea is to ensure a constant radiant intensity on an interval of orientation angles. In this case, the effect of the IRED emission pattern could be obviated from the model definition. However, this alternative would be impractical, because a new limit to the practical implementation would be added to the proposed alternative.

Considering these conditions, the camera would capture images of the IRED. From the analysis of the captured images, the relative accumulated image irradiance (*E_r_rel__*) would be estimated. The relative accumulated irradiance (*E_r_rel__*) is obtained from the inverse radiometric response function of the camera *f* using the normalized gray level intensities (*M_i_*) of the pixels *i*, *i* = 1,…, *A*, where *A* is the number of pixels of the ROI that contains the IRED image. *E_r_rel__* can be written as:

(1) $$E_{r_{\text{rel}}}=\frac{1}{A}\sum_{i=1}^{A}f\;(M_{i}\;)$$

where the fact of using normalized gray level intensities implies to divide the accumulated irradiance of pixel *i* over the maximum gray level intensity; therefore *E_r_rel__* is an dimensionless parameter.

Under the conditions stated above, *E_r_rel__* is related to the magnitudes that affect the quantity of light that falls on the sensor surface and is also accumulated by the camera. These magnitudes are the intensity radiated by the IRED (*I*_0_), the camera exposure time (*t*), the emitter orientation angle (*θ*) and the distance between the camera and the IRED (*d*). Therefore, *E_r_rel__* is defined as:

(2) $$E_{{\text{r}}_{\text{rel}}}=G\;(I_{0},t,\theta ,d\;)$$

where *G* is obtained by analyzing the behavior of *E_r_rel__* with *t*, *I*_0_, *θ* and *d*, respectively

There is a common singularity that could be interpreted as unconsidered parameter if the distance measuring alternative would be compared with typical pin-hole model. That is: the real position of the image sensor inside the camera.

The *E_r_rel__* of the incoming light was measured on the sensor surface by traveling through the optical system of the camera. However, the distance used in the modeling process was measured using an external reference. Therefore, there is an inaccuracy in the physical distance between the IRED and the image sensor and the real distance between the camera and the IRED measured externally.

Mathematically, to convert the real distance into the physical distance traveled by the incoming light, an offset displacement (*α*) might be included in the modeling process. That is: *D* = *d* − *α*, where *D* represents the real distance measured by an external reference and *d* the physical distance traveled by the incoming light. Nevertheless, without loss of generality, the quantitative evaluation of the accuracy of the distance estimation alternative can be made considering an external reference, but obviously this reference must be kept during whole implementation. It means that in the calibration and measurement processes, the same external reference must be used.

All automatic processes of the camera are disabled. The gain of the camera was fixed to the minimum value and the brightness and gamma were also fixed to a constant value during the whole implementation.

The modeling process used to obtain the function *G* was carried out empirically by measuring the individual behavior of *E_r_rel__* with *t*, *I*_0_, *θ* and *d*, respectively. From these behaviors, *G* was defined as:

(3) $$E_{r_{\text{rel}}}=G\;(I_{0},t,\theta ,d\;)=\;(\tau_{1}t+\tau_{2}\;)\times \;(\rho_{1}I_{0}+\rho_{2}\;)\times \;(\delta_{1}d^{-2}+\delta_{2}\;)\times K_{\;(\theta \;)}$$

As was explained in [8,9,11], the individual behaviors of *E_r_rel__* with the exposure time *t*, the IRED radiant intensity *I*_0_, and distance between the IRED and the camera *d* were measured. Finally, linear functions were assumed to model them. Coefficients *τ*_1_ and *τ*_2_ were used to define a linear behavior of *E_r_rel__* with *t*. Similarly, *ρ*_1_ and *ρ*_2_ were used to model the behavior of *E_r_rel__* with *I*_0_, and *δ*_1_ and *δ*_2_ were used to model the behavior of with *d*^−2^.

Nevertheless, the behavior of *E_r_rel__* with *θ* could be an arbitrary function, and it has not been included, yet; in this case, it would be considered constant (*K*_(_*_θ_*_)_), because the camera and emitter were considered aligned [8,9,11].

After elimination of the parenthesis in the Equation (3), the relative accumulated irradiance would yield:

(4) $$E_{r_{\text{rel}}}=\kappa_{1}\frac{I_{0}t}{d^{2}}+\kappa_{2}\frac{I_{0}}{d^{2}}+\kappa_{3}\frac{t}{d^{2}}+\kappa_{4}\frac{1}{d^{2}}+\kappa_{5}I_{0}t+\kappa_{6}I_{0}+\kappa_{7}t+\kappa_{8}$$

where *κ_j_* with *j* = 1,…, 8 are the model coefficients. They depend on individual coefficients used to fit the behaviors (*τ*_1_, *τ*_2_, …, *δ*_2_ and *K*_(_*_θ_*_)_) and were estimated in a calibration process considering images captured with different *t*, different *I*_0_ (different values for IRED radiant intensities were obtained by changing the emitter bias currents) and different *d*.

The acquired data was used to form a system of equations defined by:

(5) $${\text{E}}_{{\text{r}}_{\text{rel}}}=\text{H}.k$$

where, **E**_r_rel__ is the vector of relative accumulated irradiance measured in image *n*, **H** is the equation system matrix that contains the measured values of *t*, *I*_0_ and *d*, and **k** = [*κ*_1_, …, *κ*_8_]*^t^* is the unknown vector.

Therefore, from the Equation (5), k can be calculated by:

(6) $$\begin{array}{c} \text{k}=H^{+}.E_{{\text{r}}_{\text{rel}}}\\ H^{+}={\left(H.H^{\text{t}}\right)}^{-1}.H^{\text{t}} \end{array}$$

where **H**^+^ is the pseudo-inverse matrix of **H**.

Once values for the model coefficients have been calculated, the distance between the camera and the IRED can be estimated directly from Equation (4). However, a differential methodology has been proposed to estimate the distance [9]. This methodology uses at least two IRED images taken with different exposure times; in [9] it was demonstrated that better results for distance estimation were obtained with this method than with the other alternative.

The results of the measurement process suggest that the relative accumulated irradiance *E_r_rel__* could be an alternative to obtain depth when a 3-D positioning system using one camera and one IRED onboard the robot is required [8,9,11].

### 1.2. New Parameter to Be Considered to Obtain Distance between a Camera and an IRED

The procedure described in Section 1.1 was generalized in order to use other parameters extracted from the IRED image by considering only the pixel gray level intensities.

Specifically, the additional proposed parameters were the zeroth-frequency component of the FFT of the IRED image [10] and the standard deviation of gray level intensities in the region of interest containing the IRED image [12]. These were defined as alternative models and were tested in distance estimations between the camera and the IRED.

The zeroth-frequency component of the FFT of the IRED image represents the average gray level intensity. Thus, using the FFT to obtain the average gray level is an ineffective procedure. However, strategically the FFT of the IRED image will be used to obtain new parameters to be related to radiometric magnitudes including the distance between the camera and the IRED.

Nevertheless, in this paper only the zeroth-frequency component of the FFT of the image of the IRED is used for distance estimation.

In [10], it has been demonstrated that the zeroth-frequency component (*F*(0, 0)) and *E_r_rel__* have similar models, since the behaviors of *F*(0, 0) with *t*, *I*_0_ and *d* were equivalent compared with the measured behaviors of *E_r_rel__*.

In other words, the *F*(0, 0) was characterized similarly to *E_r_rel__* as was explained in Section 1.1. The behaviors of *F*(0, 0) with *t*, *d* and *I*_0_ were measured experimentally. Finally, a linear function was considered to model the behaviors of *F*(0, 0) with *t*, *I*_0_ and *d*^−2^, respectively.

Thus, considering a linear function to model the behaviors of *F*(0, 0) with *t*, *I*_0_ and *d*^−2^, the expression for *F*(0, 0) looks like Equation (3). In this case, the effect of the orientation angles was not included in the model yet, as was explained in Section 1.1 [10].

**Figure 1.**
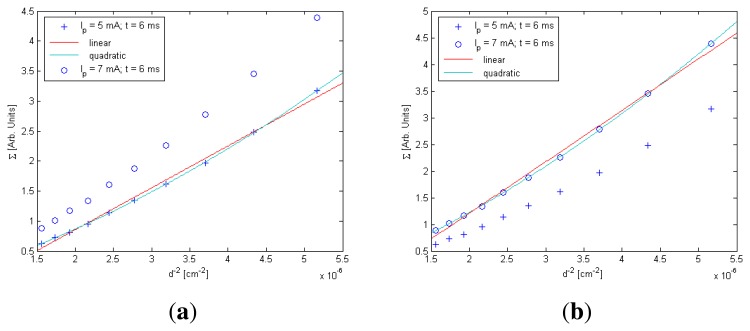
The standard deviation as a function of *d*^−2^. Using this result, a quadratic expression for the behavior of Σ with *d*^−2^ was assumed. (**a**) for *I_p_* = 5 mA and (**b**) for *I_p_* = 7 mA.

In the case of the standard deviation (Σ), the behavior of Σ with *t* and *I*_0_ was similar to *E_r_rel__* and *F*(0,0), respectively; consequently, linear behaviors were considered to model them. However, there was only a small difference in the behavior of Σ with distance compared with *E_r_rel__* and *F*(0, 0), as is shown in Figure 1 [12].

In *F*(0,0) and *E_r_rel__* models, linear functions have been used to model them with *d*^−2^. In the case of Σ, as is shown in Figure 1, the use of a quadratic function to model distance behavior will be more accurate than a linear one [12].

Finally, the additional parameters were tested to estimate the distance between one camera and one IRED, considering that the camera was aligned with the IRED. In both cases, the relative error in distance estimation was lower than 3% in a range of distances from 4 to 8 m.

Therefore, to describe the problem defined in Section 1 related to the estimation of the distance between one camera and one IRED onboard a robot, three independent alternatives have been proposed, which are summarized in Table 1.

Nevertheless, some questions remain unanswered, for example:

- What will happen when the camera and emitter are not aligned?
- How can we ensure that the estimated distance is kept constant under different conditions, e.g., for different temperatures (knowing that the IRED radiant intensity depends on temperature)?
- Can IRED radiant intensity be eliminated or estimated using the defined models?

These questions will be answered in this paper, following the principal objective, which is to propose a distance measurement methodology that provides a better performance in real environments by integrating the distance estimation alternatives summarized in Table 1.

The following sections describe the solution to the problems stated above. First, in Section 2, the effect of the IRED orientation angle is incorporated into the models summarized in Table 1, to ensure that the models can be used in non-aligned situations. Next, in Section 3, a study is presented on how the influence of IRED radiant intensity can be eliminated from distance estimation methodology. In Section 4, a distance estimation method is described, taking into account the results of the study carried out in Section 3. Section 5 describes the experiments conducted to validate the *I*_0_-free distance estimation method and the corresponding results. Finally, in Section 6, the conclusions and future trends are presented.

## 2. Introducing the Orientation Angle Effect

**Figure 2.**
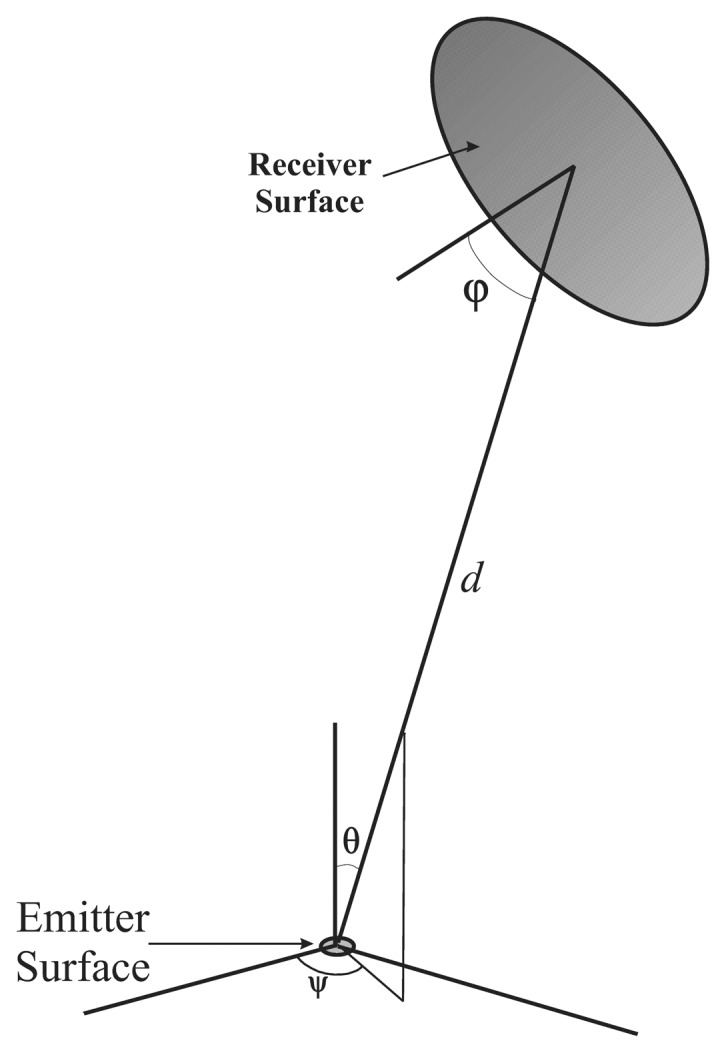
Simplified diagram of the problem of the distance estimation between a camera (Receiver Surface) and a IRED (Source).

In the measurement alternatives proposed in [8–12], the camera and the IRED were considered aligned. In practice, the IRED onboard the robot could have any orientation angle with respect to the camera. In other words, the IRED could be placed in any position in the positioning cell, and consequently the IRED image coordinates would vary, implying that different incidence angles would be obtained. Figure 2 shows a simplified diagram of the orientation angles that must be considered in the problem of estimating the distance between a camera and an IRED.

As can be seen in Figure 2, there are three angles involved into the problem of camera-to-emitter distance estimation. Two angles are related to the IRED: *ψ* and *θ*, which are used to describe the IRED emission pattern. *ψ* can be excluded because in most cases the emission pattern is a figure of revolution. Thus, the *θ* angle and the incidence angle (*φ*) are the main orientation angles that must be considered. The *θ* orientation angle is directly related to the quantity of light emitted to the camera and is also involved in the IRED emission pattern; therefore, this angle must be included in the distance estimation alternatives.

The effect of the incidence angle (*φ*) is related to the vignetting phenomenon, which produces a reduction in the gray level intensities of peripheral pixels compared with the center of the image. In addition, the incidence angle can be estimated by the geometric model when the focal distance and the optical center of the camera are known. That is, $\varphi =\tan^{-1}\frac{\left\|\vec{r}\right\|}{f}$, where ‖*r⃗*‖ represents the distance between the IRED image coordinates and the image coordinates of the optical center, and *f* is the focal distance. Nevertheless, the vignetting effect can be reduced if a telecentric lens is used.

From a radiometric point of view, the main angle to be considered in the distance estimation alternatives is the *θ* orientation angle, because it ponders the quantity of energy emitted to the camera by the emission pattern.

Although the effect of the IRED orientation angle (*θ*) was measured in [8–12], as can be seen in Table 1, it has not been included in mathematical models.

**Figure 3.**
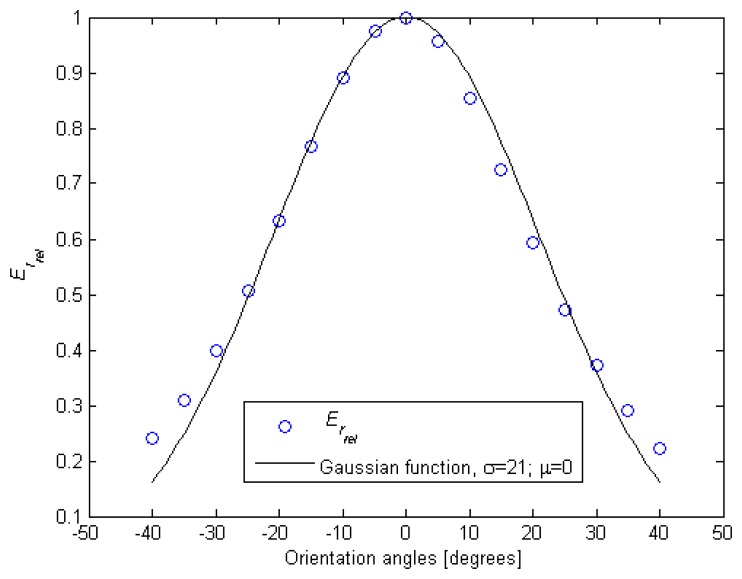
Measured behavior of *E_r_rel__* with the IRED orientation angle. A Gaussian function is used to fit this behavior.

Starting with the relative accumulated image irradiance, Figure 3 depicts the behavior of *E_r_rel__* with the IRED orientation angle (*θ*). As can be seen, this behavior can be modeled by a Gaussian function [16].

Figure 3 demonstrates the statement summarized in Table 1, namely that *E_r_rel__* can be modeled by a linear function of the function used to fit the IRED pattern.

Mathematically, to take into account the *θ* orientation angle, an additional product must be included in the Equation (3). That is:

(7) $$E_{r_{\text{rel}}}=G\;(I_{0},t,\theta ,d\;)=\;(\tau_{1}t+\tau_{2}\;).\;(\rho_{1}I_{0}+\rho_{2}\;).\;(\delta_{1}d^{-2}+\delta_{2}\;).\;(\gamma_{1}\varrho \left(\theta \right)+\gamma_{2}\;)$$

where $\varrho \left(\theta \right)=e^{\frac{1}{2}\frac{\theta^{2}}{\sigma^{2}}}$ is the Gaussian function shown in Figure 3, and (*γ*_1_ϱ + *γ*_2_) states that *E_r_rel__* is a linear function of the Gaussian function used to model the emission pattern.

Once the parentheses have been eliminated, the relative accumulated image irradiance would yield:

(8) $$E_{r_{\text{rel}},\theta }=\{\begin{array}{c} \kappa_{1}\frac{I_{0}t}{d^{2}}\varrho \left(\theta \right)+\kappa_{2}\frac{I_{0}}{d^{2}}\varrho \left(\theta \right)+\kappa_{3}\frac{t}{d^{2}}\varrho \left(\theta \right)+\kappa_{4}\frac{\varrho \left(\theta \right)}{d^{2}}+\\ \kappa_{5}I_{0}t\varrho \left(\theta \right)+\kappa_{6}I_{0}\varrho \left(\theta \right)+\kappa_{7}t\varrho \left(\theta \right)+\kappa_{8}\varrho \left(\theta \right)+\\ \kappa_{9}\frac{I_{0}t}{d^{2}}+\kappa_{10}\frac{I_{0}}{d^{2}}+\kappa_{11}\frac{t}{d^{2}}+\kappa_{12}\frac{1}{d^{2}}+\kappa_{13}I_{0}t+\kappa_{14}I_{0}+\kappa_{15}t+\kappa_{16} \end{array}$$

which represents a 16-coefficients model that can be calculated in a calibration process by considering different *t*, *d*, *I*_0_ and different IRED orientation angles.

The values for *κ_j_* with *j* = 1, …, 16 are those that minimize the error between theoretical and measured *E_r_rel__*. That is:

(9) $$\epsilon =\sum_{n=1}^{N}{\left[E_{r_{\text{rel}},\theta }^{\text{Measured}}-E_{r_{\text{rel}},\theta }^{\text{Theoret}}\;(x,k\;)\right]}^{2}$$

where **k** = [*κ*_1_, …, *κ*_16_, *σ*]*^t^* is the unknown vector. In this case, the deviation of the Gaussian function that models the emitter pattern is also included as an additional unknown to ensure the validity for others IREDs; x is the calibration data vector that contains the values of *t*, *I*_0_ and *d*, and *θ* is considered over *n* = 1, …, *N* images.

**Figure 4.**
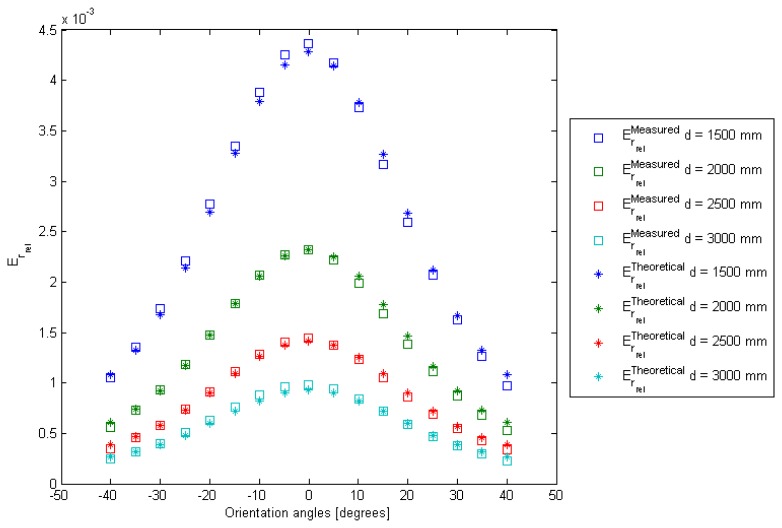
Relative accumulated image irradiance model fitting taking into account the IRED orientation angle.

The result of this fitting process is shown in Figure 4.

Figure 4 gives the result of model fitting for different distances between the IRED and the camera and for different IRED orientation angles. The relative error in this model fitting process was lower than 4%.

Once the coefficients vector **k** has been calculated, the model defined in Equation (8) can be used for distance estimation. As in [9–12], a differential methodology can be used, but an estimation of *θ* is required to calculate the camera-to-emitter distance. Thus, a methodology to estimate the IRED orientation angle outside the *E_r_rel__* model is needed in order to estimate the distance.

In [17], a method to estimate the camera pan-tilt angles by detecting circular shapes was proposed, and this idea was used to estimate the IRED orientation angle. However, in contrast to [17], in our system the camera was static and placed in a fixed position. Thus, if a circular IRED is used, the orientation angles calculated by [17] can be used in the *E_r_rel__* alternative.

As was demonstrated in [17], the pan-tilt angle of the IRED can be estimated from the estimated ellipse and its parameters: the minor and major axes and the angle formed between the horizontal axis of the image and the major axis.

**Figure 5.**
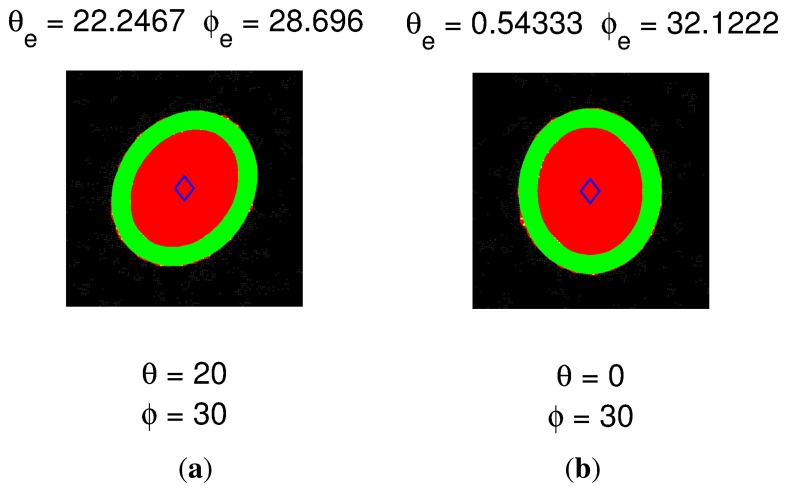
Result of IRED orientation angle estimation using the method proposed by [17]. Green points are the points that belong to the ellipse border, the red points represent all pixels of the IRED image and the blue point represents the center of the ellipse. The IRED orientation angle estimated by this method was subsequently used as the initial value for an optimization procedure, as will be explained in Section 4.

Figure 5 depicts a typical result for the IRED orientation angle. The figure shows the estimated ellipse, the center point and the points that belong to the IRED image. In Figure 5(a), the real pan-tilt angles were 20 and 30 degrees, respectively, and in Figure 5(b), the orientation angles were 0 and 30 degrees. The estimated IRED orientation angles were obtained with ±2 degrees of error and were subsequently used as the initial value in an optimization process, as will be explained in Section 4.

**Figure 6.**
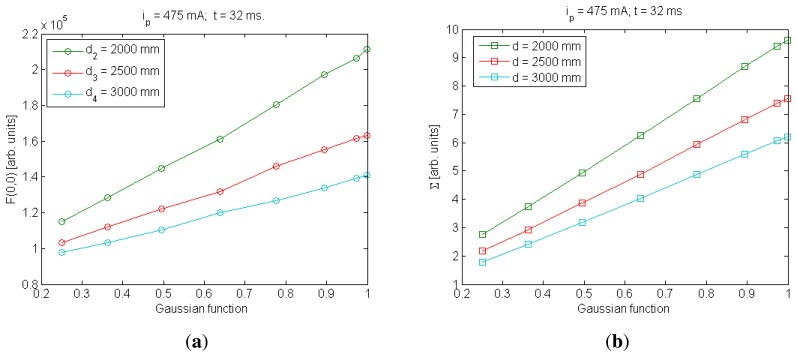
Other additional parameters extracted from IRED images and their relationship with the function used for IRED emission pattern modeling, (**a**) behavior for *F*(0,0); (**b**) behavior for Σ.

The methodology employed to include the orientation angle in the *E_r_rel__* alternative was also used to include the orientation angles in the *F*(0,0) and Σ alternatives. In both cases, their behaviors with the IRED orientation angle were considered linear, and consequently a linear product was added to their specific expression. Figure 6 shows the behavior of *F*(0,0) and Σ with a Gaussian function used to model the IRED emission pattern. Note that qualitatively, the assumption of linear functions to model the effect of the IRED emission pattern could be a valid statement.

As in the case of the *E_r_rel__* model, the calibration process also considered the Gaussian dispersion as an unknown in the *F*(0, 0) and Σ models, respectively. Table 2 summarizes the equations for *E_r_rel__*, *F*(0, 0) and Σ in the general form.

## 3. Defining a Radiant Intensity-Free Model

The other principal problem to be analyzed in this paper is the variation in IRED characteristics under different conditions, for example, temperature. As stated earlier, radiant intensity can be set by the IRED bias current. As the IRED is a semiconductor diode, temperature variation produces a drift in bias current that is transferred to radiant intensity [18] and thus affects the distance estimation.

A priori, an estimation of IRED radiant intensity would be a practical solution, but knowing the *I*_0_ would not give us any useful information related to the positioning system. The ideal solution would be to eliminate, at least in the mathematical procedure, the effect of IRED radiant intensity on the distance estimation method.

Three equations were obtained mathematically, shown in Table 2; if a system of equations could be formed using the three parameters/equations summarized in Table 2, then three unknowns could be calculated. As stated above, there is no benefit to be gained in estimating the value of *I*_0_, therefore, eliminating it would be the best solution.

First, the equations summarized in Table 2 were written in differential form as defined in [9,10,12]. This study demonstrated that a better performance is obtained than with a direct distance estimation method.

The differential method analyzes two images captured with different exposure times *t_m_* and *t_r_*, respectively. The difference of *E_r_rel__* in the two images, $\Delta E_{r_{\text{rel}}}=E_{r_{\text{rel}}}^{m}-E_{r_{\text{rel}}}^{r}$, can be defined as:

(10) $$\begin{array}{lll} \Delta E_{r_{\text{rel}}} & = & \kappa_{1}I_{0}\varrho \;(\theta \;)D\Delta t_{j}+\kappa_{3}\varrho \;(\theta \;)D\Delta t_{j}+\\ & & +\kappa_{5}I_{0}\varrho \;(\theta \;)\Delta t_{j}+\kappa_{7}\varrho \;(\theta \;)\Delta t_{j}+\\ & & +\kappa_{9}I_{0}D\Delta t_{j}+\kappa_{11}D\Delta t+\kappa_{13}I_{0}\Delta t_{j}+\kappa_{15}\Delta t_{j} \end{array}$$

where $\Delta t_{j}=t_{m}-t_{r},E_{r_{\text{rel}}}^{m}$ is the relative accumulated image irradiance in the image captured with *t_m_* exposure and $E_{r_{\text{rel}}}^{r}$ represents the *E_r_rel__* for the image captured with *t_r_*. The *t_r_* time is called the reference exposure time.

Analogously, for the *F*(0, 0) parameter:

(11) $$\begin{array}{ll} \Delta F{\left(0,0\right)}_{j}= & \beta_{1}I_{0}\varrho_{F}\;(\theta \;)D\Delta t_{j}+\beta_{3}\varrho_{F}\;(\theta \;)D\Delta t_{j}+\\ & +\beta_{5}I_{0}\varrho_{F}\;(\theta \;)\Delta t_{j}+\beta_{7}\varrho_{F}(\theta \;)\Delta t_{j}+\\ & +\beta_{9}I_{0}D\Delta t_{j}+\beta_{11}D\Delta t_{j}+\beta_{13}I_{0}\Delta t_{j}+\beta_{15}\Delta t_{j} \end{array}$$

Finally, for Σ:

(12) $$\begin{array}{lll} \Delta \sum_{j} & = & \lambda_{1}I_{0}\Delta t_{j}D^{2}\varrho_{\sum }\;(\theta \;)+\lambda_{2}I_{0}\Delta t_{j}D\varrho_{\sum }\;(\theta \;)+\lambda_{3}I_{0}\Delta t_{j}\varrho_{\sum }\;(\theta \;)+\\ & & +\lambda_{4}\Delta t_{j}D^{2}\varrho_{\sum }\;(\theta \;)+\lambda_{5}\Delta t_{j}D\varrho_{\sum }\;(\theta \;)+\lambda_{6}\Delta t_{j}\varrho_{\sum }\;(\theta \;)+\\ & & +\lambda_{13}\Delta t_{j}I_{0}D^{2}+\lambda_{14}\Delta t_{j}I_{0}D+\lambda_{15}\Delta t_{j}I_{0}+\\ & & +\lambda_{16}\Delta t_{j}D^{2}+\lambda_{17}\Delta t_{j}D+\lambda_{18}\Delta t_{j} \end{array}$$

Subsequently, from Equation (11), *I*_0_ can be written as:

(13) $$I_{0_{j}}=\frac{\Delta F\;(0,0\;)-[\beta_{3}\varrho_{F}\;(\theta \;)D\Delta t_{j}+\beta_{7}\varrho_{F}\;(\theta \;)\Delta t_{j}+\beta_{11}D\Delta t_{j}+\beta_{15}\;\Delta t_{j}]}{\beta_{1}\varrho_{F}\;(\theta \;)D\Delta t_{j}+\beta_{5}\varrho_{F}\;(\theta \;)\Delta t_{j}+\beta_{9}D\Delta t_{j}+\beta_{13}\Delta t_{j}}$$

To simplify the mathematical expressions, this notation was used:

(14) $$I_{0_{j}}=\Omega \left(\varrho_{\text{F}}\;(\theta \;),\Delta t_{j},D,\Delta F\;(0,0\;),\vec{\beta }\right)$$

where *β⃗* is the vector of *β*'s coefficients from Equation (11).

The idea was to substitute *I*_0_ in the other equations, which would produce two *I*_0_-free equations.

Thus, substituting *I*_0_ from Equation (13) in Equations (10) and (12) would yield:

(15) $$\begin{array}{lll} \Delta E_{r_{\text{rel}}} & = & \kappa_{1}\left[\Omega \left(\varrho_{\text{F}}\;(\theta \;),\Delta t_{j},D,\Delta F\;(0,0\;),\vec{\beta }\right)\right]\varrho \;(\theta \;)D\Delta t_{j}+\kappa_{3}\varrho \;(\theta \;)D\Delta t_{j}+\\ & & +\kappa_{5}\left[\Omega \left(\varrho_{F}\;(\theta \;),\Delta t_{j},D,\Delta F\;(0,0\;),\vec{\beta }\right)\right]\varrho \;(\theta \;)\Delta t_{j}+\kappa_{7}\varrho \;(\theta \;)\Delta t_{j}+\\ & & +\kappa_{9}\left[\Omega \left(\varrho_{F}\;(\theta \;),\Delta t_{j},D,\Delta F\;(0,0\;),\vec{\beta }\right)\right]D\Delta t_{j}+\kappa_{11}\;D\Delta t_{j}+\\ & & +\kappa_{13}\left[\Omega \left(\varrho_{F}\;(\theta \;),\Delta t_{j},D,\Delta F\;(0,0\;),\vec{\beta }\right)\right]\Delta t_{j}+\kappa_{15}\;\Delta t_{j} \end{array}$$

for the Δ*E_r_rel__* model. In the case of ΔΣ, the *I*_0_-free equation can be written as:

(16) $$\begin{array}{lll} \Delta \Sigma & = & \lambda_{1}\left[\Omega \left(\varrho_{F}\;(\theta \;),\Delta t_{j},D,\Delta F\;(0,0\;),\vec{\beta }\right)\right]\Delta t_{j}D^{2}\varrho_{\Sigma }\;(\theta \;)+\\ & & +\lambda_{2}\left[\Omega \left(\varrho_{F}\;(\theta \;),\Delta t_{j},D,\Delta F\;(0,0\;),\vec{\beta }\right)\right]\Delta t_{j}D\varrho_{\Sigma }\;(\theta \;)+\\ & & +\lambda_{3}\left[\Omega \left(\varrho_{F}\;(\theta \;),\Delta t_{j},D,\Delta F\;(0,0\;),\vec{\beta }\right)\right]\Delta t_{j}\varrho_{\Sigma }\;(\theta \;)+\lambda_{4}\Delta t_{j}D^{2}\varrho_{\Sigma }\;(\theta \;)+\\ & & +\lambda_{5}\Delta t_{j}D\varrho_{\Sigma }\;(\theta \;)+\lambda_{6}\Delta t_{j}\varrho_{\Sigma }\;(\theta \;)+\lambda_{13}\Delta t_{j}\left[\Omega \left(\varrho_{F}\;(\theta \;),\Delta t_{j},D,\Delta F\;(0,0\;),\vec{\beta }\right)\right]D^{2}+\\ & & +\lambda_{14}\Delta t_{j}\left[\Omega \left(\varrho_{F}\;(\theta \;),\Delta t_{j},D,\Delta F\;(0,0\;),\vec{\beta }\right)\right]D+\\ & & +\lambda_{15}\Delta t_{j}\left[\Omega \left(\varrho_{F}\;(\theta \;),\Delta t_{j},D,\Delta F\;(0,0\;),\vec{\beta }\right)\right]+\lambda_{16}\Delta t_{j}D^{2}+\lambda_{17}\Delta t_{j}D+\lambda_{18}\Delta t_{j} \end{array}$$

Eliminating the parentheses in Equations (15) and (16), respectively, yields:

$$\Delta E_{r_{\text{rel}}}=\frac{\Delta E_{\text{Num}}}{\Delta E_{\text{Denom}}}$$

(17) $$\begin{array}{lll} \Delta E_{\text{Num}} & = & q_{1}\Delta F\;(0,0\;)\Delta tD\varrho_{E}\;(\theta \;)+q_{2}\Delta t^{2}D^{2}{\varrho^{2}}_{E}\;(\theta \;)+\\ & & +q_{3}\Delta t^{2}D{\varrho^{2}}_{E}\;(\theta \;)+q_{4}\Delta t^{2}D^{2}\varrho_{E}\;(\theta \;)+\\ & & +q_{5}\Delta t^{2}D\varrho_{E}\;(\theta \;)+q_{6}\Delta F\;(0,0\;)\Delta t\rho_{E}\;(\theta \;)+\\ & & +q_{7}\Delta t^{2}{\varrho^{2}}_{E}\;(\theta \;)+q_{8}\Delta t^{2}\varrho_{E}\;(\theta \;)+\\ & & +q_{9}\Delta F\;(0,0\;)\Delta tD+q_{10}\Delta t^{2}D^{2}+\\ & & +q_{11}\Delta t^{2}D+q_{12}\Delta F\;(0,0\;)\Delta t+q_{13}\Delta t^{2} \end{array}$$

$$\Delta E_{\text{Denom}}=q_{14}\Delta tD\varrho_{E}\;(\theta \;)+q_{15}\Delta t\varrho_{E}\;(\theta \;)+q_{16}\Delta tD+q_{17}\Delta t$$

for the model of relative accumulated image irradiance. The coefficients *q_j_* with *j* = 1, …, 17 are the model coefficients that were estimated in a calibration process where *σ_E_* was also taken into account, which is the dispersion of the Gaussian function used to model the IRED emission pattern.

The function ϱ*_E_* in Equation (17) is formed by the product of ϱ, which is the Gaussian function used to model the IRED emission pattern in the *E_r_rel__* model, and ϱ*_F_* is an equivalent function for the *F*(0, 0) model: the resulting function remains a Gaussian function with a different dispersion. As was explained in Section 2, in the calibration process the Gaussian dispersion parameter was considered as an additional unknown to guarantee the best possible model fitting.

In the case of the standard deviation of pixel gray level intensities in the ROI containing the IRED image, the *I*_0_-free expression would yield:

$$\Delta \sum^{\text{Indp}}=\frac{\Delta \sum_{\text{Num}}}{\Delta \sum_{\text{Denom}}}$$

(18) $$\begin{array}{lll} \Delta \sum_{\text{Num}} & = & p_{1}\Delta F\;(0,0\;)\Delta tD^{2}\varrho_{\sum }\;(\theta \;)+p_{2}\Delta t^{2}D^{3}\varrho_{\Sigma }^{2}\;(\theta \;)+\\ & & +p_{3}\Delta t^{2}\varrho_{\Sigma }^{2}D^{2}+p_{4}\Delta t^{2}D^{3}\varrho_{\Sigma }\;(\theta \;)+\\ & & +p_{5}\Delta t^{2}D^{3}\varrho_{\Sigma }\;(\theta \;)+p_{6}\Delta F\;(0,0\;)\Delta tD\varrho_{\Sigma }\;(\theta \;)+\\ & & +p_{7}\Delta t^{2}D\varrho_{\Sigma }^{2}\;(\theta \;)+p_{8}\Delta t^{2}D\varrho_{\Sigma }\;(\theta \;)+\\ & & +p_{9}\Delta F\;(0,0\;)\Delta t\varrho_{E}\;(\theta \;)+p_{10}\Delta t^{2}\varrho_{\Sigma }^{2}\;(\theta \;)+\\ & & +p_{11}\Delta t^{2}\varrho_{\Sigma }\;(\theta \;)+p_{12}\Delta F\;(0,0\;)\Delta tD^{2}+\\ & & +p_{13}\Delta t^{2}D^{3}+p_{14}\Delta t^{2}D^{2}+p_{15}\Delta F\;(0,0\;)\Delta tD+\\ & & +p_{16}\Delta t^{2}D+p_{17}\Delta F\;(0,0\;)\Delta t+p_{18}\Delta t^{2} \end{array}$$

$$\Delta \Sigma_{\text{Denom}}=p_{19}\Delta tD\varrho_{\Sigma }\;(\theta \;)+p_{20}\Delta t\varrho_{\Sigma }\;(\theta \;)+p_{21}\Delta tD+p_{22}\Delta t$$

where *p_j_* with *j* = 1, …, 22 are the model coefficients. For this case, 22 + 1 unknowns were considered, because the dispersion of the Gaussian function used to model the IRED emitter pattern was introduced as an additional unknown, as stated in Equation (17).

Formally, the model fitting process for Equations (17) and (18) would be written as:

(19) $${ɛ}_{E}\;(\text{q}\;)=\sum_{n=1}^{N}{\left[\Delta E_{r_{\text{rel}}}^{\text{Measured}}-\Delta E_{r_{\text{rel}}}^{\text{Theoret}}\;({\text{x}}_{n},\text{q}\;)\right]}^{2}$$

where **x***_n_* represents the model fitting data, which takes into account *n* = 1, …, *N* images captured with different exposure times, IRED radiant intensities, IRED orientation angles and IRED-to-camera distances. Vector q = [*q*_1_, …, *q*_1_7, *σ_E_*] represents the vector of unknowns. Note that the dispersion of the Gaussian function used to model the IRED emitter pattern was included as an additional unknown in the model fitting process. *∊_E_* is the error between measured and theoretical *E_r_rel__*.

Equation (19) describes the calibration process for the Δ*E_r_rel__* model. In the case of ΔΣ, the model fitting process can be defined as:

(20) $${ɛ}_{E}\;(\text{p}\;)=\sum_{n=1}^{N}{\left[\Delta \Sigma_{n}^{\text{Measured}}-\Delta \Sigma_{n}^{\text{Theoretical}}\;({\text{x}}_{n},\text{p}\;)\right]}^{2}$$

where **p** = [*p*_1_, …, *p*_22_, *σ*_Σ_] represent the vectors that are unknown, with an additional unknown, as was considered in Equation (19)**x***_n_* represents the same set of calibration data used in Equation (19) and *∊*_Σ_ is the error between measured and theoretical Σ.

In Equations (19) and (20), an optimization process was carried out to obtain the vectors **q** and **p**, which minimize the errors *∊_E_* and *∊*_Σ_ respectively, using the Levenberg-Marquardt algorithm.

**Figure 7.**
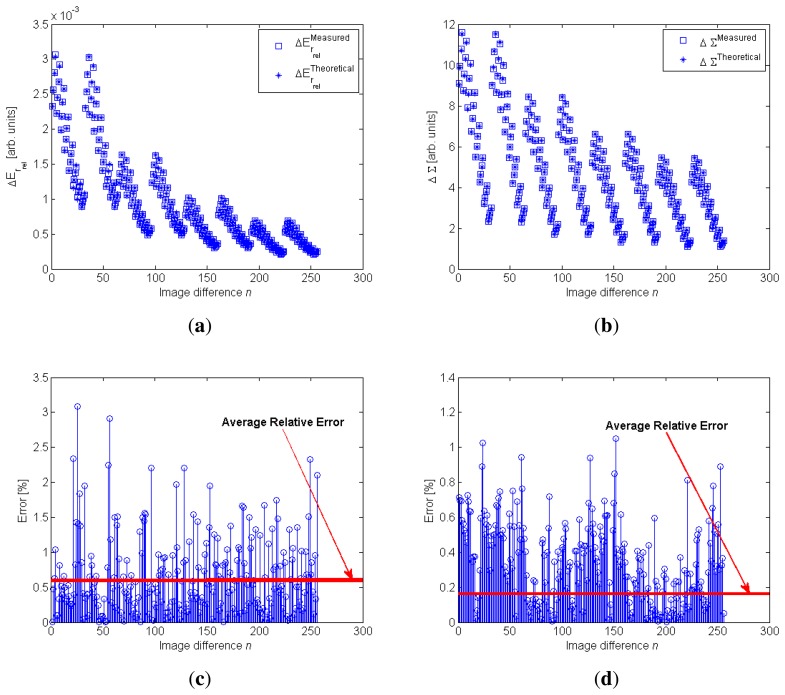
Results for model fitting considering the Equations (19) and (20). The fitting data x*_n_* were composed of images captured with different exposure times, IRED orientation angles, distances and IRED radiant intensities. (**a,b**) are the model fitting for the *E_r_rel__* model and the ΔΣ model, respectively; (**c,d**) are the relative errors in [%] for model fitting for Δ*E_r_rel__* and ΔΣ, respectively; in addition, the average relative error is also shown.

The results for the model fitting process are shown in Figure 7.

In Figure 7(a,b) the blue square represents the measured Δ*E_r_rel__* and ΔΣ values and the asterisk represents the theoretical values. Each point shown in Figure 7(a,b) represents the differences of *E_r_rel__* and Σ of two images captured with different exposure times.

The results shown in these figures demonstrate that the defined models can be considered valid to mathematically characterize the Δ*E_r_rel__* and ΔΣ parameters; this validity has also been demonstrated by the errors of the model fitting process, which are shown in Figure 7(c,d). Specifically, for the ΔΣ model, the fitting errors were lower than the Δ*E_r_rel__* model, but in both cases the average relative error considering all image differences was lower than 1%.

## 4. Estimating the Distance between the Camera and the IRED Independently of IRED Radiant Intensity

The measurement alternative is formed by the Equations (17) and (18), which are independent of IRED radiant intensity *I*_0_.

Once the models coefficients q and p have been calculated, for each model, an expression to estimate the distance can be defined. For example, for the Δ*E_r_rel__* model, the distance would yield:

(21) $$a_{1}D^{2}+a_{2}D+a_{3}=0$$

where the *a*_1, 2, 3_ coefficients were defined as:

(22) $$\begin{array}{c} a_{1}=q_{2}\Delta t^{2}{\varrho^{2}}_{E}\;(\theta \;)+q_{4}\Delta t^{2}\varrho_{E}\;(\theta \;)+q_{10}\Delta t^{2}\\ \begin{array}{ll} a_{2}= & q_{1}\Delta F\;(0,0\;)\Delta t\varrho_{E}\;(\theta \;)+q_{3}\Delta t^{2}{\varrho^{2}}_{E}\;(\theta \;)+q_{5}\Delta t^{2}\varrho_{E}\;(\theta \;)+q_{9}\Delta F\;(0,0\;)\Delta t+\\ & +q_{11}\Delta t^{2}-q_{14}\Delta E_{r_{\text{rel}}}\Delta t\varrho_{E}\;(\theta \;)-q_{16}\Delta E_{r_{\text{rel}}}\Delta t \end{array}\\ \begin{array}{ll} a_{3}= & q_{6}\Delta F\;(0,0\;)\Delta t\varrho_{E}\;(\theta \;)+q_{7}\Delta t^{2}{\varrho^{2}}_{E}\;(\theta \;)+q_{8}\Delta t^{2}\varrho_{E}\;(\theta \;)+q_{12}\Delta F\;(0,0\;)\Delta t+\\ & +q_{13}\Delta t^{2}-q_{15}\Delta E_{r_{\text{rel}}}\Delta t\varrho_{E}\;(\theta \;)-q_{17}\Delta E_{r_{\text{rel}}}\Delta t \end{array} \end{array}$$

Analogously, for the ΔΣ model:

(23) $$c_{1}D^{3}+c_{2}D^{2}+c_{3}D+c_{4}=0$$

and the coefficient of Equation (23) can be obtained by:

(24) $$\begin{array}{c} c_{1}=p_{2}\Delta t^{2}\varrho_{\sum }^{2}\;(\theta \;)+p_{4}\Delta t^{2}\varrho_{\sum }\;(\theta \;)+p_{13}\Delta t^{2}\\ c_{2}=p_{1}\Delta F\;(0,0\;)\Delta t\varrho_{\sum }\;(\theta \;)+p_{3}\Delta t^{2}\varrho_{\sum }^{2}\;(\theta \;)+p_{5}\Delta t^{2}\varrho_{\sum }\;(\theta \;)+p_{12}\Delta F\;(0,0\;)\Delta t+p_{14}\Delta t^{2}\\ \begin{array}{ll} c_{3}= & p_{6}\Delta F\;(0,0\;)\Delta t\varrho_{\sum }\;(\theta \;)+p_{7}\Delta t^{2}\varrho_{\sum }^{2}\;(\theta \;)-p_{19}\Delta \Sigma \Delta \varrho_{\sum }\;(\theta \;)-p_{21}\Delta \Sigma \Delta t+\\ & +p_{8}\Delta t^{2}\varrho_{\sum }\;(\theta \;)+p_{16}\Delta t^{2} \end{array}\\ \begin{array}{ll} c_{4}= & p_{9}\Delta F\;(0,0\;)\Delta t\varrho_{\sum }\;(\theta \;)+p_{10}\Delta t^{2}\varrho_{\sum }^{2}\;(\theta \;)+p_{11}\Delta t^{2}\varrho_{\sum }\;(\theta \;)+p_{17}\Delta F(0,0\;)\Delta t+\\ & +p_{18}\Delta t^{2}-p_{20}\Delta \Sigma \Delta t\varrho_{\sum }\;(\theta \;)-p_{22}\Delta \Sigma \Delta t \end{array} \end{array}$$

The coefficients *a*_1, 2, 3_ and *c*_1, 2, 3, 4_ in Equations (22) and (24) depend on Δ*E_r_rel__* Δ*F*(0, 0), ΔΣ, which are extracted from images, Δ*t*, which is measured in the camera, and *θ*, which is calculated by the estimated ellipse using the method proposed in [17] and also explained in Section 2. Therefore, once the coefficients in Equations (22) and (24) have been estimated, Equations (21) and (23) can be solved to obtain the distance between the camera and the IRED.

From the root of each of these two equations, the Equations (21) and (23) provide the distance estimation. The contribution of each of these equations could be weighted to take accuracy in the model fitting process into account, as was proposed in [2]. However, in this case the weighting contribution was not considered. Consequently, Equations (21) and (23) were equaled. Thus, the distance can be calculated by solving the following equation:

(25) $$c_{1}D^{3}+\;(c_{2}-a_{1}\;)D^{2}+\;(c_{3}-a_{2}\;)D+\;(c_{4}-a_{3}\;)=0$$

where the distance *d* would be $d=+\sqrt{D}$, and *D* is the positive and real root of the Equation (25).

Mathematically, Equations (21) and (23) have at least one equal roots. If both equations are equaled, then the intersection points of both equations could be estimated. As was shown in Equation (25), the intersection of Equations (21) and (23) is defined by a cubic equation. By obtaining the roots of Equation (24), the solution for the intersection of Equations (21) and (23) can be estimated.

**Figure 8.**
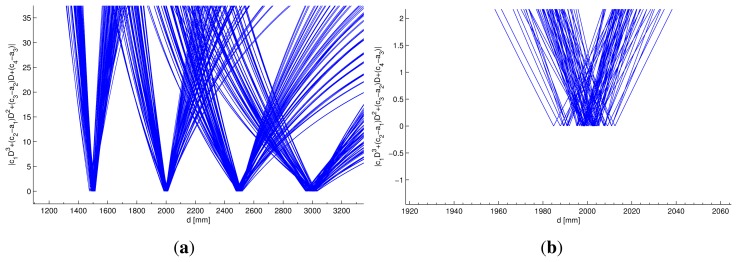
Function |*c*_1_*D*^3^ + (*c*_2_ − *a*_1_) *D*^2^ + (*c*_3_ − *a*_2_) *D* + (*c*_4_ − *a*_3_)| using the data employed in the calibration process. (**a**) The real and positive roots of Equation (25) coincide with real distances used in the calibration process (1,500, 2,000, 2,500 and 3,000 mm); (**b**) results of the roots near 2,000 mm. Note that the dispersion in the estimated distance using the calibration data is lower than 2% of the real distance value.

Figure 8 was generated from the data used in the calibration process. It graphically shows that the roots of Equation (25) correspond to the real distance employed to obtain the model coefficients.

Figure 8 shows the behavior of Equation (25) in the range of distances from 1,000 to 3,000 mm for all conditions taken into account in the calibration process.

Each condition was formed by the combination of the values of *I*_0_, *d*, *θ* and Δ*t* used in the calibration process. For each of the conditions, a set of values for *a*_1,2,3_ and *c*_1,2,3,4_ coefficients can be obtained, so Equation (25) would be different for each of the conditions used in the calibration process. Specifically, Figure 8 was formed by superposing the entire set of Equation (25) obtained using the calibration data.

Although the data used in the calibration process will be formally defined in Table 3 in Section 5, it can be observed in Figure 8 that Equation (25) has zeros closer to the real distance value used in the calibration process, with dispersion lower than 2% of the real distance value.

Using the fact that two *I*_0_-free equations have been defined, another additional unknown was considered to form a system of two equations with two unknowns. Thus, the goal was to calculate the distance between the camera and the IRED as well as the IRED orientation angle simultaneously. Mathematically, this can defined as:

(26) $$\{\begin{array}{c} \Delta E_{r_{\text{rel}}}^{\text{Measured}}-\Delta E_{r_{\text{rel}}}^{\text{Therotical}}\;(\text{x},{\text{Y}}_{{\text{E}}_{r_{\text{rel}}}}\;)=0\\ \Delta \sum^{\text{Measured}}-\Delta E^{\text{Therotical}}\;(\text{x},{\text{Y}}_{\sum }\;)=0 \end{array}$$

where the goal is to apply an optimization method to obtain the vector **x** = [*d*, *θ*]*^t^*, which minimizes the error between measured and theoretical values, where **Y_Errel_** and **Y_∑_** are the vectors that contain the *p* and *q* coefficients, the Δ*t* values and the values for Δ*F*(0, 0). The optimization method requires an initial estimation for **x**, and this was considered as the *θ* value obtained from the estimated ellipse and the distance obtained by Equation (25).

Evidently, using the system of equations defined in Equation (26) constitutes a more general solution to the problem of estimating the distance between a camera and an IRED by considering only the information extracted for pixel grey level intensities and camera exposure times. Furthermore, by estimating from Equation (26) the distance and angle of orientation of the IRED, more efficient use is made of the possibilities offered by this system of equations.

The final alternative proposed in this paper uses the optimization stated in Equation (26) and summarized in Algorithm 1.

**Algorithm 1** Distance Measurement Alternative.
**Input:** One image of the IRED captured with a reference exposure time *t_r_* and *n* = 1, …, *N* images of the IRED captured with different exposure times *t_n_*.**Output:** Distance between the IRED and the camera *d*^(n)^ and the IRED orientation angle *θ*^(^*^n^*^)^.1:Estimating parameters from image: 
$\left(E_{r_{\text{rel}}}^{\;(r\;)},F{\left(0,0\right)}^{\;(r\;)},\sum^{\;(r\;)}\right)\leftarrow \text{Imag}e_{\;(r\;)}$2:**for**
*n* = 1 → *N*
**do**3: Δ*t*^(^*^n^*^)^ = *t_n_* − *t_r_*4: Estimating parameters from image: 
$\left(E_{r_{\text{rel}}}^{\;(n\;)},F{\left(0,0\right)}^{\;(n\;)},\sum^{\;(n\;)}\right)\leftarrow \text{Imag}e_{\;(n\;)}$5: Forming the differences: 
$\{\begin{array}{l} \Delta E_{r_{\text{rel}}}^{\;(n\;)}=E_{r_{\text{rel}}}^{\;(n\;)}-E_{r_{\text{rel}}}^{\;(r\;)}\\ \Delta F{\left(0,0\right)}^{\;(n\;)}=F{\left(0,0\right)}^{\;(n\;)}-F{\left(0,0\right)}^{\;(r\;)}\\ \Delta \sum^{\;(n\;)}=\sum^{\;(n\;)}-\sum^{\;(r\;)} \end{array}$6: Estimating the IRED orientation angle: 
$\theta_{0}^{\;(n\;)}\leftarrow \text{Imag}e_{\;(n\;)}$ by method of the estimated ellipse [17].7: Calculating the coefficients *a*^(^*^n^*^)^ and *c*^(^*^n^*^)^ from Equations (22) and (24), respectively.8: Obtaining the initial distance estimation 
$d_{0}^{\;(n\;)}$ by solving *c*_1_*D*^3^ + (*c*_2_ − *a*_1_)*D*^2^ + (*c*_3_ − *a*_2_)*D* + (*c*_4_ − *a*_3_) = 09: Optimization method to calculate simultaneously *d*^(^*^n^*^)^ and *θ*^(^*^n^*^)^10:
$\{\begin{array}{c} \Delta E_{r_{\text{rel}}}^{\text{Measured}}-\Delta E_{r_{\text{rel}}}^{\text{Theoretical}}\;(\text{x},{\text{Y}}_{{\text{E}}_{r_{\text{rel}}}}\;)=0\\ \Delta \Sigma^{\text{Measured}}-\Delta \Sigma^{\text{Theoretical}}\;(\text{x},{\text{Y}}_{\sum }\;)=0 \end{array}$ with 
$x_{0}={\left[d_{0}^{\;(n\;)},\theta_{0}^{\;(n\;)}\right]}^{t}$ as initial estimations.11:**end for**


Another interesting aspect addressed in this paper, which can be inferred from Algorithm 1, is that different exposure times were considered to test the alternative for estimating the distance and the IRED orientation angle. This is related to the fact analyzed in [19]. In this study, a differential method to estimate the distance between an IRED and a camera was proposed using the relative accumulated image irradiance. The novelty of this approach resides in the selection of optimum exposure times to perform the distance measurement process. These optimum exposure times can be determined from the *bathtub curves*, which were obtained from the relative error in the model fitting process. That is why in Algorithm 1, *n* = 1, …, *N* exposure times were considered in order to obtain elements from which to select the optimum exposure times to execute the distance estimation algorithm.

The algorithm for estimating the distance and the IRED orientation angle uses several images captured with different exposure times *t_n_* and one image captured with *t_r_*, which is a reference exposure time, to obtain the differences Δ*t*^(^*^n^*^)^, $\Delta E_{r_{\text{rel}}}^{\;(n\;)}$, Δ*F*(0,0)^(^*^n^*^)^ and ΔΣ^(^*^n^*^)^. Using the images captured with *t_n_* exposure times, the *θ*^(^*^n^*^)^ were estimated from the estimated ellipse, as proposed in [17]. With the calculated differences and the IRED orientation angle estimated by [17], the coefficients *a* and *c* can be calculated. This permits the initial distance estimation to be obtained. Finally, the initial distance estimation obtained by solving Equation (25) and the initial IRED orientation angle obtained by [17] were used as initial values to solve the system of equations, Equation (26). The resultant x from Equation (26) constitutes the final distance and IRED orientation angle estimation.

## 5. Experimental Setup, Results and Discussions

The main goal of the experimental tests was to demonstrate that the alternatives defined in Equations (17) and (18) and summarized in Algorithm 1 are independent of IRED bias current. Therefore, the term *independent* means that the measurement alternative is independent of the IRED radiant intensity.

**Figure 9.**
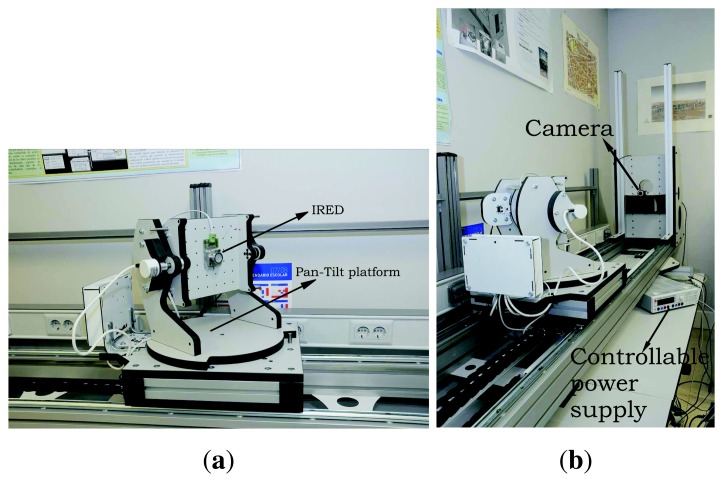
Measurement station used in practical validation of the alternative to measure the distance between the camera and the IRED. (**a**) Pan-tilt platform with the circular IRED attached; (**b**) Platform shown from another angle to depict the camera.

The tests were carried out using the measurement station shown in Figure 9.

To ensure real distances and IRED orientation, an accurate ad-hoc measurement station was built, which was controlled from a PC by serial port communication. The measurement station was composed of a pan-tilt platform onto which the IRED was mounted and which permitted the IRED orientation angles to be changed with a precision of 0.01 degrees. As can be seen in Figure 9(b), the measurement station also allowed the distance between the camera and the pan-tilt platform to be changed with a precision of 0.01 mm.

Figure 9 also shows the camera, the IRED and the power supply to bias the IRED.

It was necessary to calibrate the distance estimation alternative before the distance estimation process could begin. In other words, the model coefficient values had to be calculated before initiating the distance estimation process.

The calibration process was described briefly in Section 3, to demonstrate the validity of the defined model, as shown in Figure 7. However, the data used to estimate the coefficients' values are summarized in Table 3.

By combining the conditions summarized in Table 3, namely, four distances, two IRED bias currents, eight IRED orientation angles and five exposure times, a dataset of 320 images was obtained. From the analysis of these images, 256 parameter differences were obtained, which means that 256 equations were defined. As indicated in Section 3, the results of this calibration process are shown in Figure 7.

**Figure 10.**
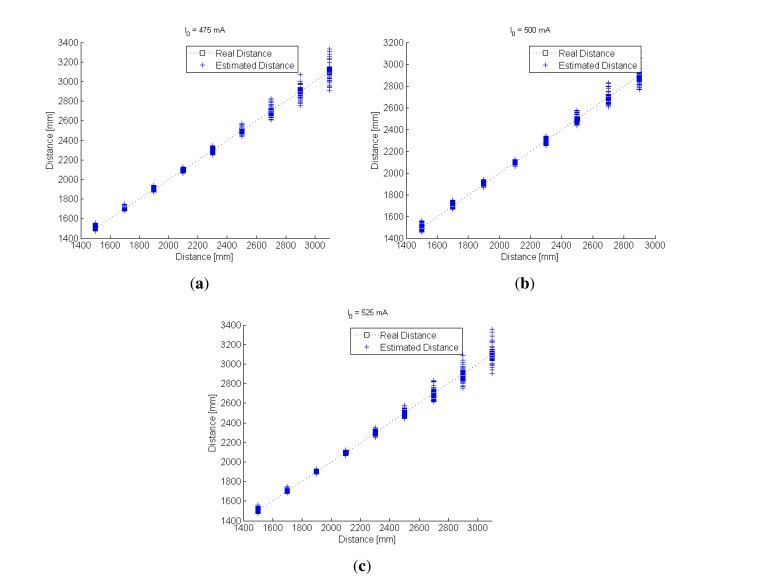
Results of the distance estimation process considering all available Δ*t* and all IRED orientation angles. (**a**) for a bias current of 475 mA, (**b**) for 500 mA and (**c**) for 525 mA.

Once the model coefficients had been calculated, an experiment to estimate the distance between the IRED and the camera was carried out. In this experiment, a reference exposure time *t_r_* =8 ms was considered and images were captured with exposure times from *t_n_* = 30 to 40 ms in 2 ms steps. With these times, the differences in exposure times (Δ*t_n_*) would yield: Δ*t_n_* = 22, 24, …, 32 ms. The distance was varied from 1,500 to 2,900 mm in 20 cm steps, and the IRED orientation angles were fixed at 0, 10, 20 and 30 degrees. For all available Δ*t_n_*, a distance estimation was obtained by using the Equation (25). In addition, the IRED bias currents were set at 475, 500 and 525 mA. The results are shown in Figure 10. The goal of this experiment was to test independence from *I*_0_ in the distance estimation process, considering all possible conditions. Furthermore, it should be noted that in this experiment, a new value for *I*_0_ was used, which had not been considered in the calibration process.

In Figure 10, all distance estimations for all available Δ*t* and for all IRED orientation angles for the three different bias currents are superposed. In the three cases (Figure 10), the estimated distances were qualitatively similar. This indicates that the influence of IRED radiant intensity variation was considerably minimized. However, the accuracy of the distance estimations depends on the accuracy of IRED angle estimation and the differences in exposure times used in the distance measurement process.

**Figure 11.**
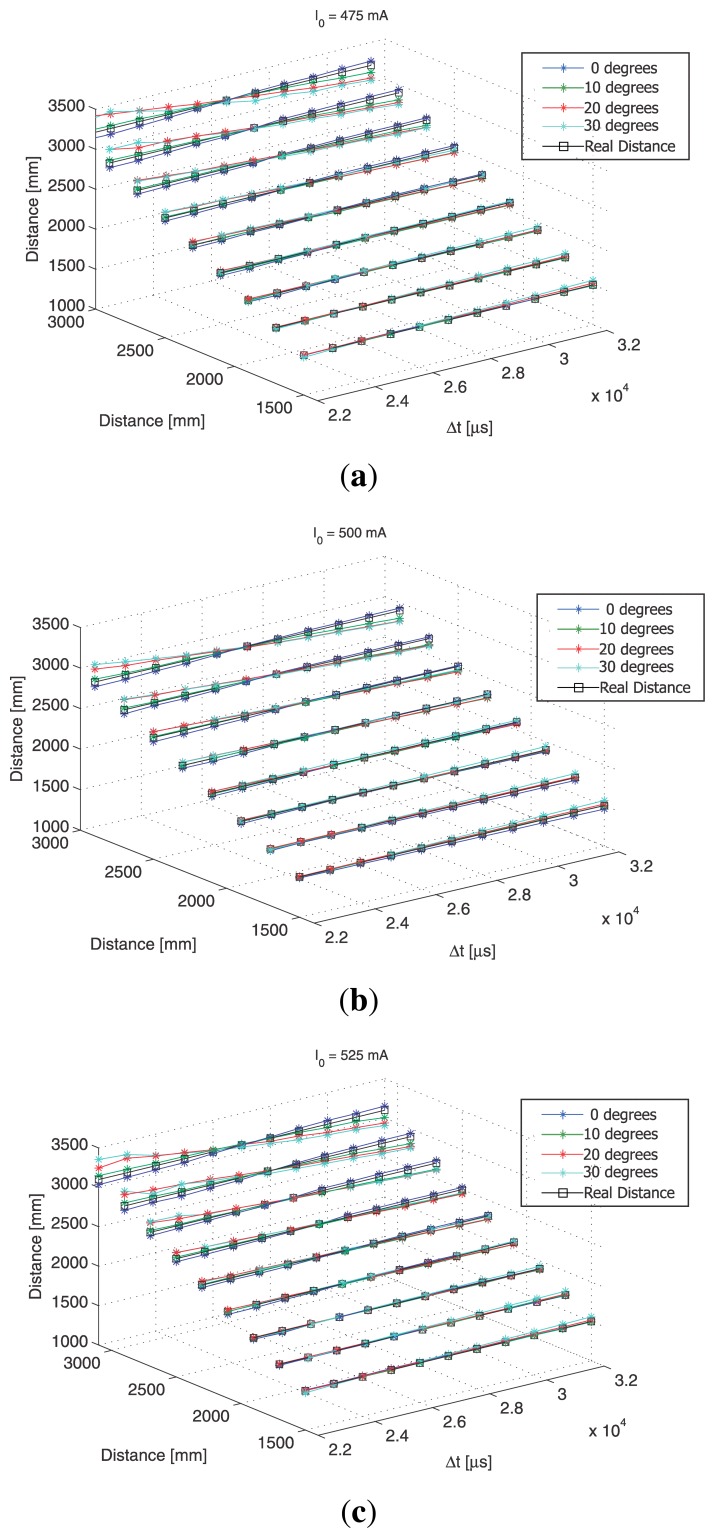
Results of distance estimation process as a function of Δ*t* for all IRED orientation angles considered. (**a**) for a bias current of 475 mA, (**b**) for 500 mA and (**c**) for 525 mA.

To clarify the relationship between the IRED orientation angles and different exposure times, the data plotted in Figure 10 is represented in a 3-D space, as shown in Figure 11, with the goal of determining the influence of differences in exposure times on the distance estimation process.

The dependency of distance estimation accuracy on the differences in exposure times was analyzed in [19], who demonstrated that there is an optimum exposure time difference where the best accuracy in distance estimation can be achieved. Lázaro *et al* [19] proposed estimating the optimum measurement conditions from the calibration process. In this case, the optimum exposure time difference was estimated by an analysis of the distance estimations shown in Figure 10.

**Figure 12.**
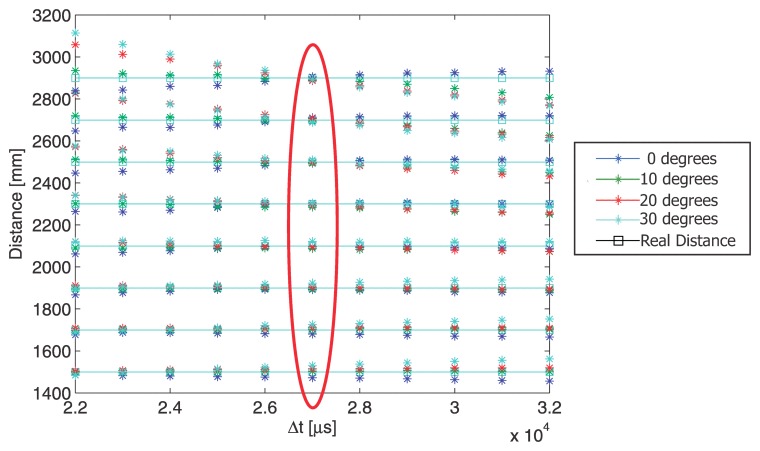
Distance estimation as a function of Δ*t*. Note that best accuracy was obtained with Δ*t* = 27 ms. Consequently, this was the optimal exposure time difference for the distance estimation process.

Thus, by analyzing Figure 11, it was possible to determine the exposure time difference where best accuracy would be obtained. In this case, the experimental results indicated that best accuracy in distance and IRED orientation angle estimations was obtained using Δ*t* = 27 ms, as can be seen in Figure 12.

**Figure 13.**
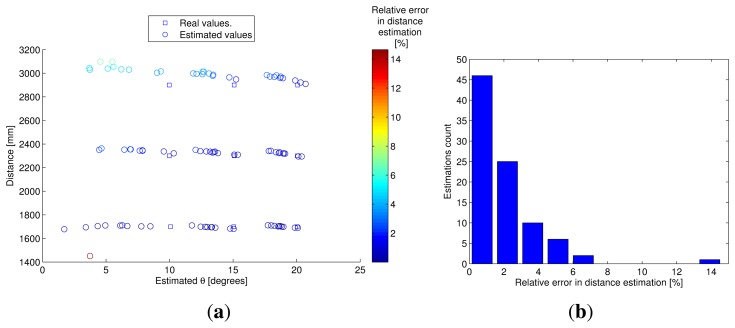
Results of the distance and IRED orientation angle estimation using random bias currents in the IRED. (**a**) Distance and IRED orientation angle estimation. The (*x*, *y*) coordinates represent the estimated *θ* and *d*, respectively. The squares are the true coordinates used in the experiment and the circles are the estimated values. The color of the circles represents the relative error in [%] of the estimated distance. (**b**) Histogram of relative errors in distance estimations.

Using the optimum exposure time difference, another experiment was carried out to test the alternative for estimating the distance between the IRED and the camera and the IRED orientation angle simultaneously, as described in Algorithm 1. This experiment considered distances of 1,700, 2,300 and 2,900 mm between the camera and the IRED, and 10, 15 and 20 degrees for the IRED orientation. Besides, unlike the previous experiment, the IRED was biased with a random bias current with a mean of 500 mA and a dispersion of 50 mA. A random value for the IRED bias current was generated for each distance–orientation pair and for each *d*–*θ* pair: ten random bias currents were used to bias the emitter and to capture images to estimate the distance and the IRED orientation. In total, 90 *d*–*θ* pairs were estimated. The results of the *d*– *θ* pairs are shown in Figure 13.

In Figure 13, the blue square represents the real *d*–*θ* pairs and the colored circle represents the estimated *d*–*θ* pairs. The (*x*, *y*) coordinates of these points represent the IRED orientation angle and the distance, respectively. The color of the circles encodes the relative error in distance estimation.

There is an interesting aspect that merits emphasis, which is related to the accuracy of distance estimations. Accuracy can be obtained from Figure 13, from the difference between *y* coordinates for real and estimated points. For these cases, the error was lower than the differences between *x* coordinates, which represent the error of IRED orientation angles estimations. Therefore, even with an IRED random bias current, the proposed measurement alternative maintained less than 2% relative error throughout the majority of distance estimations. Figure 13(b) was constructed precisely in order to summarize the behavior of the relative error for all the estimated distances. This figure is a histogram of all relative errors in distance estimations and it can be concluded that from the 90 estimations, the relative error in more than 50% of these was lower than 2%. Indeed, this figure was closer to 1% in approximately 46 estimations, which represented 51% of the distance estimations.

However, as is shown in Figure 13(a), estimation of the IRED orientation angle was not as accurate in this experiment as the distance estimation. In fact, better estimations of the IRED orientation angles would have been obtained using the estimated ellipse method proposed in [17].

Although estimation of the IRED orientation angle was not as accurate as distance estimation, the results obtained were qualitatively accurate, especially for estimated distance. Furthermore, the worst estimations for the IRED orientation angles were obtained for lower than 10 degrees, as shown in Figure 13. In a practical positioning system, the camera must be mounted so as to ensure the greatest field of vision, in order to cover a larger area; the most common solution is to tilt the camera. This means that the optical axes of the camera form an angle with the vertical direction. On the other hand, the IRED is oriented towards the ceiling, and thus the angle between the IRED maximum direction and the vertical direction is zero. In this case, and in most IRED positions, the IRED orientation angle would be higher than 10 degrees and could be estimated by the geometrical model.

**Figure 14.**
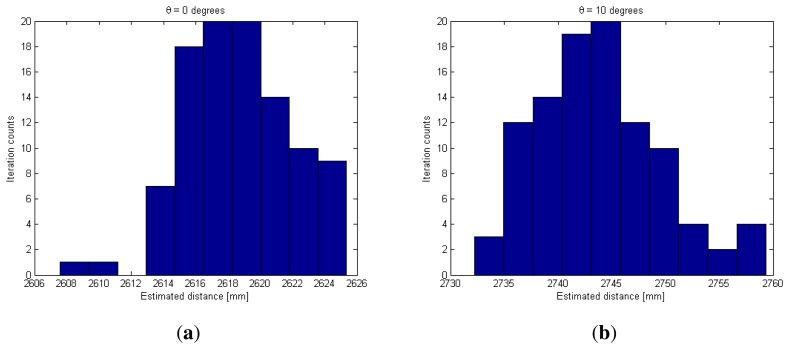

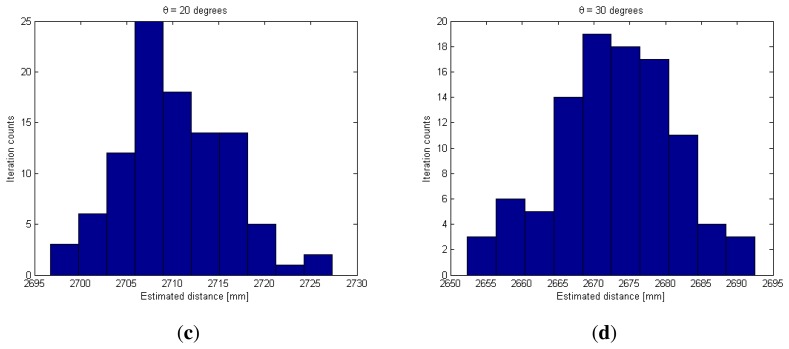
Consistency of the distance estimation alternative proposed in this paper. (**a**) Considering *θ* = 0 degrees; (**b**) Considering *θ* = 10 degrees; (**c**) Considering *θ* = 20 degrees; (**d**) Considering *θ* = 30 degrees.

As a final experiment, the consistency of the distance measurement alternative was tested. The measurement alternative summarized in Algorithm 1 was included in a loop of 100 iterations. In each iteration, two images were captured, one with *t_r_* = 8 ms and the other with *t_n_* = 35 ms, with a fixed IRED bias current. The 100 distance estimations for a distance of 2,700 mm and for 0, 10, 20 and 30 degrees of IRED orientation angle are shown in Figure 14. The estimated IRED orientation angles were also estimated but more importance was given to distance estimation.

From Figure 14, the dispersion in distance estimation can be calculated. Note that the proposed alternative provides less than 3% accuracy for distance estimation with 15 mm of dispersion in the range of distance from 1 m to 3 m.

As a final comment, one aspect that requires further study is the selection of the magnitudes included in the camera-IRED system, especially the IRED bias current and the camera exposure times.

The values used in this paper were selected based on empirical results; therefore, the automatic selection of these values would improve the calibration and measurement process.

## 6. Conclusions and Future Work

In this paper, an IRED radiant-intensity-free model has been proposed to decouple the intensity radiated by the IRED from the distance estimation method using only the information extracted from pixel gray level intensities and a radiometric analysis of the image formation process in the camera.

The camera-to-emitter distance estimation alternatives proposed in [8,10–12] are dependent on IRED radiant intensity, which constitutes a drawback for future implementation using the abovementioned alternatives individually. As was explained earlier, IRED radiant intensity depends on the IRED bias current and this varies according to many factors, including temperature. In this particular case, temperature drifts would be transferred to the distance estimations. This imposes the requirement that the *I*_0_ parameter must be decoupled from the camera-to-IRED distance estimation alternatives.

The previously proposed alternatives to estimate the distance between an IRED and a camera have also considered the camera as aligned with the IRED, which reduces the possibilities of future implementations. However, in this paper, a study of the effect of the IRED orientation angle was performed and this effect was modeled by a Gaussian function.

The dispersion of the Gaussian function used to model the effect of the IRED emission pattern was included as an additional unknown in the calibration process for each individual model defined for camera-to-IRED distance estimation. In the model validation, it has been demonstrated that a maximum error of 4% was obtained for the three individual models proposed in [8,10–12].

Once the IRED orientation angle effect had been described mathematically, a method to estimate the IRED orientation angle was implemented by using a circular IRED through the estimation of the ellipse formed by the projection of the circular IRED on the image plane. This method provided an IRED orientation angle with 2 degrees of maximum error.

However, the main contribution of this paper is the mathematical approach to characterizing the IRED—camera system independently of IRED radiant intensity, by using the models defined in [8,10–12] together in order to form a system of equations.

The procedure to eliminate the effect of IRED radiant intensity uses the *F*(0, 0) model to obtain an expression for *I*_0_, which is substituted in the *E_r_rel__* and Σ models, respectively. Therefore, two *I*_0_-free expressions were obtained.

From the two *I*_0_-free expressions, the distance is the main unknown; however, an optimization scheme was defined to calculate the distance between the IRED and the camera and the IRED orientation angle simultaneously.

The *I*_0_-free alternative was tested for distance estimation in the range from 1,500 to 2,900 mm with three different IRED bias currents, for different IRED orientation angles and different exposure times. The results of distance estimations were very similar for all the conditions considered in this experiment. Furthermore, the experimental results obtained permitted the selection of optimum exposure time differences where distance estimations were more accurate.

Once the optimum exposure times had been selected, another experiment was carried out. In this case, the IRED was biased using a random bias current, in order to determine the independence of the distance estimation method from variations in IRED radiant intensity. Considering the random bias current, the experimental results demonstrated that the proposed alternative provided a maximum error of 2% in distance estimation. However, the IRED orientation angle estimations were not as accurate as the distance estimations.

As a further validation experiment, the consistency of the distance estimation method was tested for three different distance values over 100 repetitions. The results over the 100 repetitions showed that the maximum error of the average distance estimation was lower than 3% and the maximum dispersion was lower than 15 mm.

Finally, some aspects require further research. For example, throughout the modeling process, the IRED radiant intensity and the camera exposure times were selected empirically. Thus, a quality index based on on-line analysis of the IRED images acquired by the camera, to facilitate the assignment of values for these magnitudes, must be defined in order to increase the efficiency of the modeling and measurement processes, respectively.

## Figures and Tables

**Table 1.** Summary of parameter behaviors used to define the proposed models for camera-to-IRED distance estimation method. The final expression for each model can be obtained by the product of functions defined in each column. For example Σ = (*τ*_Σ1_*t* + *τ*_Σ2_) × (*ρ*_Σ1_*I*_0_ + *ρ*_Σ2_) × (*δ*_Σ1_*D*^2^ + *δ*_Σ2_*D* + *δ*_Σ3_) × (*γ*_Σ1_*f*(*θ*) + *γ*_Σ2_).

**Model Parameter**	***f*(*t*)**	***f*(*I*_0_)**	***f*(*d*^−2^)**	***f*(*θ*)**
*E_r_rel__*	Linear	Linear	Linear	Linear with a function of the emitter pattern
*F*(0, 0)	Linear	Linear	Linear	Linear with a function of the emitter pattern
Σ	Linear	Linear	Quadratic	Linear with a function of the emitter pattern

**Table 2.** Summary of parameters extracted from the IRED images, including the effect of the IRED orientation angle. *D* = *d*^−2^ and $\varrho_{x}\left(\theta \right)=e^{\frac{1}{2}\frac{\theta^{2}}{\sigma_{x}^{2}}}$; the *x* represents the model parameter *E_rrel_F*(0, 0) or Σ.

**Parameter**	**Equation in the General Form**
*E_r_rel__*	(*τ*_*E*_1__ *t* + *τ*_*E*_2__) × (*ϱ*_*E*_1__ *I*_0_ + *ϱ*_*E*_2__) × (*δ*_*E*_1__ *D* + *δ*_*E*_2__) × (*γ*_*E*_1__ ϱ_*E*_(*θ*) + *γ*_*E*_2__)
*F*(0, 0)	(*τ*_*F*_1__ *t* + *τ*_*F*_2__) × (*ρ*_*F*_1__ *I*_0_ + *ρ*_*F*_2__) × (*δ*_*F*_1__ *D* + *δ*_*F*_2__) × (*γ*_*F*_1__ *ϱ_F_* (*θ*) + *γ*_*F*_2__)
Σ	(*τ*_Σ_1__ *t* + *τ*_Σ_2__) × (*ρ*_Σ_1__ *I*_0_ + *ρ*_Σ_2__) × (*δ*_Σ_1__ *D*^2^ + *δ*_Σ_2__ *D* + *δ*_Σ_3__) × (*γ*_Σ_1__ *ϱ*_Σ_ (*θ*) + *γ*_Σ_2__)

**Table 3.** Calibration Data.

**Calibration Data**	
Exposure time [ms]	*t_r_* =8, *t_n_* =30, 32, 34 and 36
IRED bias current [mA]	475 and 500
Distances [mm]	1500, 2000, 2500 and 3000
IRED orientation angles [degrees]	0, 5, 10, 15, 20, 25, 30 and 35
